# Epicatechin Protects against Corticosteroid Induced Hepatic Steatosis

**DOI:** 10.4172/2157-7536.1000122

**Published:** 2014-01-02

**Authors:** Jeffrey Thomas, Miguel A Lanaspa, George Schreiner, Richard J Johnson

**Affiliations:** Division of Nephrology, University of Colorado, Aurora CO 80045, USA

Epicatechin, the flavonol derived from chocolate, can improve endothelial function, decrease inflammation and potentially improve insulin resistance [1]. It has also been shown to improve skeletal muscle and hepatic AMPK activity in diabetic mice [2]. Recently our group and others have shown that the stimulation of hepatic AMPK can block hepatic steatosis from fructose [3] or high fat diet [4]. This led us to hypothesize that epicathecin will also have a beneficial role in reducing steroid-induced fatty liver by a similar activation of AMPK.

Hepatic steatosis was induced in male Wistar rats with daily subcutaneous injections of prednisolone (10 mg/kg) for 7 days. Prednisolone induced a catabolic state, with a decrease in body weight of 26% (±1.9%) (vs. weight gain in controls of 7%, n=6 per group). Despite this weight loss, prednisolone treated rats developed fasting hyperglycemia (231 mg/dL vs. 116 in controls, p<0.05), hypertriglyceridemia (241 mg/dL vs. 119 in controls, p<0.05) and elevated intrahepatic triglycerides (131.8 mg/dL normalized for protein content vs. 87.3 mg/dl in controls, p<0.05). Rats given epicathecin (1 mg/kg via gavage twice daily, n=6) prior to and during glucocorticoid administration developed similar hyperglycemia (206 ± 48.6 mg/dL), but had a reduction in both serum and intrahepatic (IHTG) triglycerides (TG- 241 ± 100.8 vs. 164.9 ± 100.8, NS; IHTG- 147.6 ± 52.6 vs. 86.9 ± 32.4, p=0.043, for control versus epicatechin-treated rats, respectively).

Western blot of hepatic enzymes involved in *de novo* lipogenesis (acetyl CoA carboxylase, ATP citrate synthase lyase and fatty acid synthase) demonstrated no significant changes in protein levels between groups, suggesting that the changes in fatty liver induced by glucocorticoids likely results from increased activity in these enzymes or a reduction in fatty acid oxidation (data not shown). Phosphorylated AMPK was decreased with prednisolone treatment compared to control animals but was restored by epicatechin (Figure 1A, all Western blots controlled with beta-actin) perhaps as a result of increased AMP to ATP ratio. In addition beta-hydroxybutyrate, a ketone indicative of fat oxidation was blunted in glucocorticoid treated animals, with improvement among animals receiving epicathecin (beta-hydroxybutyrate levels: control 1.86 ± 0.76 nmol/mg of triglyceride vs. pred 0.65 ± 0.08 vs. epi 0.91 ± .15, p<0.05 between all groups). Liver tissues stained for fat with Oil red O staining also showed an improvement in epicatechin treated animals (Figure 1B). These studies show that epicatechin may be another therapy that can stimulate AMPK, resulting in increased fat oxidation and the prevention of hepatic steatosis.

## Figures and Tables

**Figure 1A:**
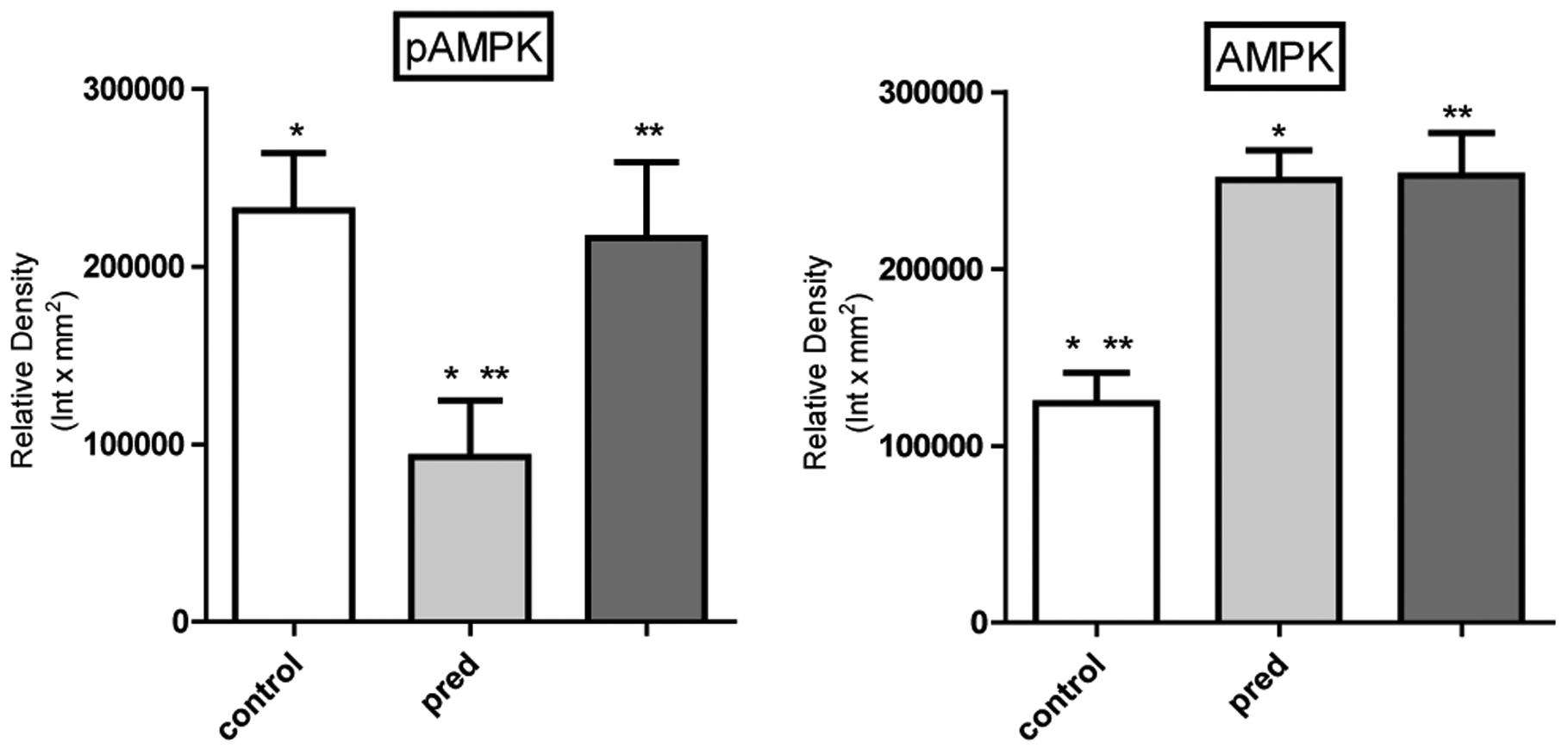
Western blot densitometry from all rats analyzed of activated and total AMP Kinase (pAMPK, AMPK), *p<0.01, **p<0.05 (n=6 animals per group).

**Figure 1B:**
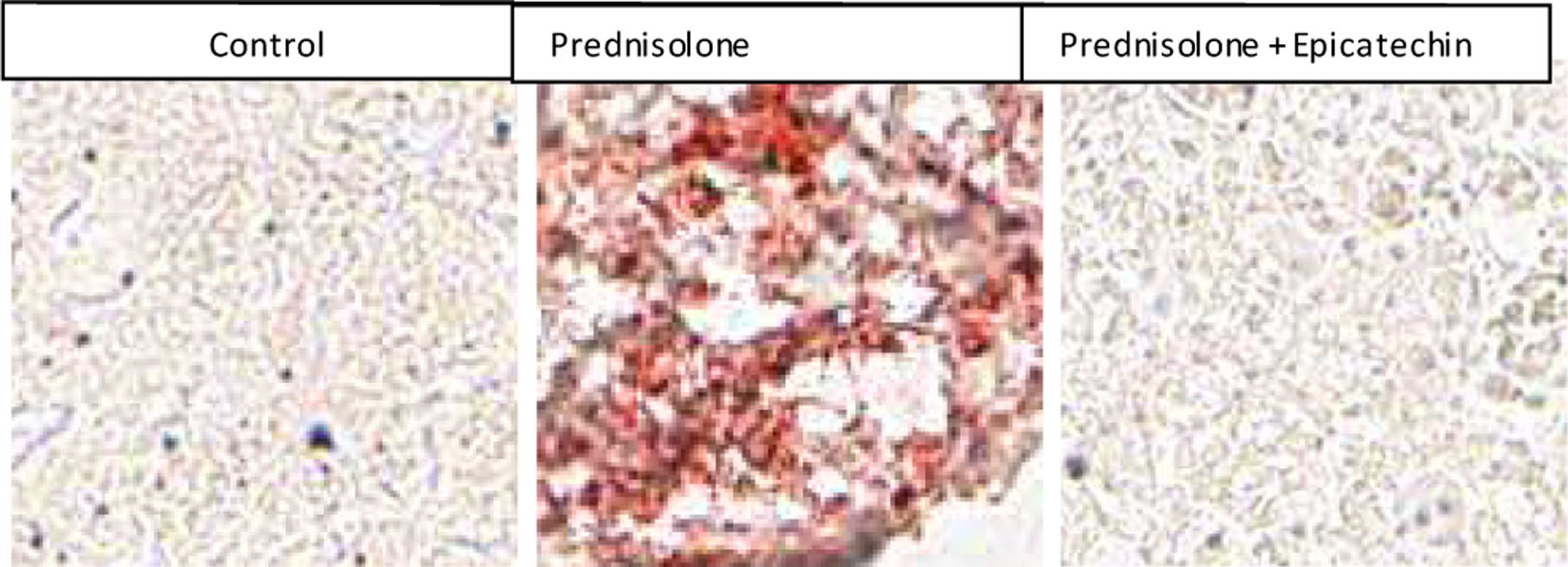
Representative liver sections stained with Oil Red.
